# Correlation between dietary score and depression in cancer patients: Data from the 2005–2018 National Health and Nutrition Examination Surveys

**DOI:** 10.3389/fpsyg.2022.978913

**Published:** 2022-09-16

**Authors:** Nan Xu, Qing An

**Affiliations:** ^1^Department of Traditional Chinese Medicine, Jiangsu Cancer Hospital and Jiangsu Institute of Cancer Research, The Affiliated Cancer Hospital of Nanjing Medical University, Nanjing, China; ^2^Nanjing University of Chinese Medicine, Nanjing, China

**Keywords:** cancer, dietary score, depression, NHANES database, association

## Abstract

**Aim:**

To investigate the correlation between dietary score and depression in patients with cancer.

**Methods:**

In this cross-sectional study, data were collected from the National Health and Nutrition Examination Survey (NHANES) 2005–2018, a cross-sectional and nationally representative database, to compare 322 patients with depression to 2,868 with no depression. Mediterranean (MEDS) diet, Dietary Approaches to Stop Hypertension (DASH) diet, and the Healthy Eating Index 2015 (HEI-2015) score were calculated. Depression was assessed using the Patient Health Questionnaire-9 (PHQ-9). Weighted logistic regression models were used to explore the relationship between dietary scores and depression in patients with cancer. Subgroup analysis was performed by sleep disorders, sex, cancer type, number of tumors, and pain relief prescription treatment.

**Results:**

The final study sample included 3,190 adults, with 56.94% of them being women, representing 2,177 (86.51%) non-Hispanic white adults. After multivariable adjustment, the MEDS score was correlated with a reduced risk of depression in patients with cancer [odds ratio (OR): 0.90, 95% confidence interval (CI): 0.82–0.97, *p* = 0.010]. Moreover, the MEDS score was correlated with depression in cancer patients with sleep disorders (OR: 0.84, 95% CI: 0.76–0.93, *p* = 0.001), in female patients with cancer (OR: 0.83, 95% CI: 0.74–0.92, *p* < 0.001), particularly in female cancer reproductive system patients (OR: 0.69, 95% CI: 0.57–0.82, *p* < 0.001). MEDS score also showed a decreased risk of depression in patients with 1 cancer (OR: 0.90, 95% CI: 0.82–0.98, *p* = 0.019). MEDS score (OR: 0.86, 95% CI: 0.86–0.98, *p* = 0.024) and DASH (OR: 0.91, 95% CI: 0.84–0.98, *p* = 0.015) score were related to a decreased risk of depression in patients with cancer using pain relief prescription.

**Conclusion:**

Good diet quality is significantly correlated with decreased risk of depression in patients with cancer. Aligning with the Dietary Guidelines, such as the MEDS diet, may be beneficial to the reduced risk of depression in patients with cancer.

## Introduction

Cancer is a major public health problem worldwide and is the second leading cause of death in the United States (Viale, 2020; Siegel et al., 2021). Although substantial improvements have been made in the clinical treatment of cancer, its overall prognosis remains unfavorable (Miller et al., 2019). As cancer has a substantial effect on patients' physical appearance, physical ability, family, occupation, economic status, and emotions during diagnosis and treatment (Le et al., 2018), depression becomes a common psychological disease that is comorbid with cancer (Akimana et al., 2019). Depression damages the immune system and promotes the progression of cancer, which leads to worse disease outcomes (Robson et al., 2017). Depression also increases patient treatment non-compliance, such as failure to attend consultation appointments, and leads to poorer treatment response (Erim et al., 2019). A better understanding of the influencing factors of depression among patients with cancer may not only prolong the lives of patients but also improve their quality of life.

Existing evidence based on the National Health and Nutrition Examination Survey (NHANES) has assessed the relationship between overall health and diet (Glover et al., 2022a). Several epidemiological studies have found a correlation between individual food types and nutrients (such as folic acid or omega-3 fatty acids) and the risk of depression (Sakai et al., 2017; Li et al., 2018; Rahimlou et al., 2018). While most of them focused on specific food intake (Lee et al., 2021; Steele et al., 2021). Many researchers also found that adherence to specific dietary patterns, such as the Mediterranean (MEDS) diet (Sánchez-Villegas et al., 2013), Dietary Approaches to Stop Hypertension (DASH) diet (Perez-Cornago et al., 2017), and the Healthy Eating Index 2015 (HEI-2015) score (Wang et al., 2021), was correlated with lower depression risk in non-cancer populations. However, there is extremely limited evidence about the correlation between diet scores and depression among patients with cancer. In order to alleviate the psychological stress of patients with cancer and reduce the impact of depression on the prognosis of patients with cancer, it is necessary to explore the correlation between dietary scores and depression in patients with cancer.

Herein, we conducted this study to explore whether the diet scores correlate with depression among patients with cancer and possibly provide dietary measures to reduce the risk of depression, improving the quality of life of patients with cancer.

## Methods

### Study design and participants

In this cross-sectional study, data were collected from the NHANES between 2005 and 2018. NHANES is a nationally representative, population-based survey for assessing adult and child health and nutritional status in the US. This survey combined health interviews conducted in respondents' homes with health measurements performed at mobile exam centers (MECs). The examination components consisted of medical, dental, and physiological measurements, and laboratory tests were supervised by trained medical personnel. Furthermore, the adoption of various modern equipment enabled the NHANES to collect reliable and high-quality data (Belladelli et al., 2021). Moreover, compensation and a report of medical findings were given to each participant, which increased the compliance of participants (National Health and Nutrition Examination Survey, 2020). This study included seven NHANES survey cycles (2005–2006, 2007–2008, 2009–2010, 2011–2012, 2013–2014, 2015–2016, and 2017–2018). Patients with no dietary information or a total intake of 0, patients with non-cancer, and patients with incomplete depression-related questionnaires were excluded. The sample consisted of 3,190 patients with a history of cancer. Only publicly available data were used in the study, and no ethical approval was needed in this study.

### Definition and assessments

Recommendations from MEDS included (1) abundant use of olive oil for cooking and dressing; (2) increased consumption of fruit, vegetables, legumes, and fish; (3) reduction in total meat consumption and recommending white meat instead of red or processed meat; (4) preparation of homemade sauce with tomato, garlic, onion, and spices with olive oil to dress vegetables, pasta, rice, and other dishes; (5) avoidance of butter, cream, fast food, sweets, pastries, and sugar-sweetened beverages; and (6) for alcohol drinkers, moderate consumption of red wine (Sánchez-Villegas et al., 2013). The DASH-style dietary pattern includes reducing sodium and increasing potassium intake; moderating of alcohol intake; and increasing physical activity (American Diabetes Association., 2019). The HEI is a measure for assessing dietary quality, precisely, the degree to which a set of foods aligns with the Dietary Guidelines for Americans. The HEI-2015 components were the same as in the HEI-2010, except saturated fat and added sugars replaced empty calories, with the result being 13 components (Krebs-Smith et al., 2018). HEI-2015 scores ranged from 0 to 100, with higher HEI scores reflecting better diet quality. We utilized the total nutrient intakes on the first day (DR1TOT) to calculate the 13 components of HEI-2015. For further weighted Scott-Rao chi-square test and weighted logistic regressions, HEI-2015 scores of less than 50, between 50 and 70, and more than 70 were categorized as inadequate, average, and optimal, respectively (De La Cruz et al., 2021).

Depression was assessed using the Patient Health Questionnaire-9 (PHQ-9). The PHQ-9 is a well-validated (Cronbach's α = 0.89), self-report instrument that assesses depression symptoms (i.e., sadness, trouble sleeping, fatigue, and problems concentrating) in the past 2 weeks and has moderate concordance with clinical psychiatric interviews. The PHQ-9 questionnaire contains nine items, with each item being assessed on a four-point Likert scale, ranging from 0 = not at all to 3 = nearly every day, and summing up a total scale range of 0–27. A dichotomous variable indicating no depression (PHQ-9 score <10) or increased depressive symptoms (PHQ-9 score ≥ 10) was created using a threshold score of 10 (Kroenke et al., 2001).

### Potential covariates

Potential covariates included age, sex (male and female), race (Mexican American, Other Hispanic, non-Hispanic white, non-Hispanic black, and other race, such as multi-racial), marital status (married, widowed, divorced/separated, and spinsterhood), body mass index (BMI) status (underweight and normal, overweight, and obesity), property income ratio (PIR), education status [ ≤ 11th grade, i.e., 12th grade with no diploma, high school graduate/general equivalency diploma (GED), some college or AA degree, and above], sleep disorder (yes and no), hypertension (yes and no), diabetes (yes and no), smoking status (yes and no), pain relief prescription use (yes and no), cancer types (breast cancer, cervix cancer, bladder cancer, colon cancer, kidney cancer, melanoma, ovary cancer, prostate cancer, skin cancer, and uterus cancer), and the number of tumors. PIR was categorized as ≤ 130%, >130–350%, and >350% by the ratio of family income to poverty (FPL).

### Statistical analysis

Measurement data were expressed as mean ± standard error (SE), and comparisons between different groups were performed by Student's *t*-test. Classification variables were recorded as case numbers and percentages and compared between groups using a weighted chi-square test. NHANES data had a multi-stage stratified probability sampling design. The missing values were filled by multiple interpolations. The missing values before and after interpolation were compared between groups as sensitivity analysis.

A weighted between-group difference analysis was performed in a sample population with or without depression. We then fitted multivariable weight-adjusted logistic regression models to assess the relationship between dietary score and depression in patients with cancer across three models: model 1 was an unadjusted model; model 2 was adjusted for sex, age, and race; and model 3 was adjusted for all variables with statistical differences in univariate analysis. Subgroups of the population were analyzed based on the presence or absence of sleep disorders, sex, cancer of the female reproductive system, the number of tumors, and whether pain relievers were used.

Statistical analysis was performed using a two-sided test, and statistical significance was defined as *p* < 0.05. The annual percentage changes (APCs) were calculated using Rv3.6.3 (Institute for Statistics and Mathematics, Vienna, Austria), and trend tests were performed by using the Joinpoint Program 4.0.4. The Mecweight, strata (KSTRATA), and cluster (primary sampling unit, PSU) were used for weighted analysis. All other analyses were performed using SASv9.4 (SAS Institute Inc., Cary, NC, USA).

## Results

### Characteristics of included study

**Figure 1:**
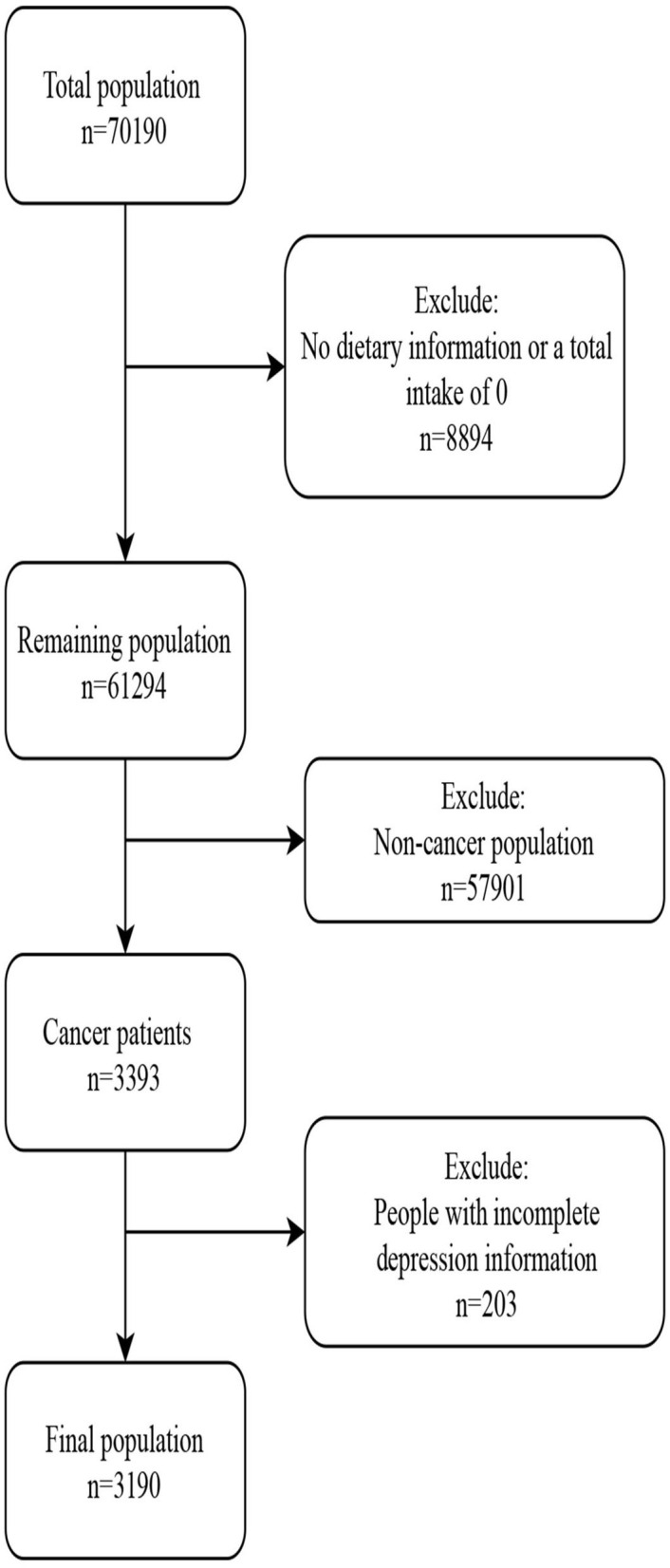
Flow chart of data collection and population inclusion process.

**Figure 2:**
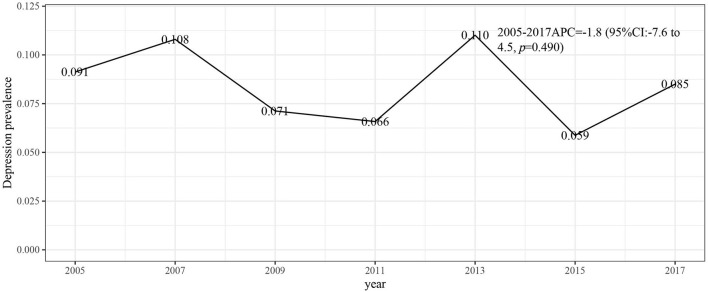
Depression annual prevalence trend between 2005 and 2017. APC, annual percentage changes; CI, confidence interval.

**Figure 3:**
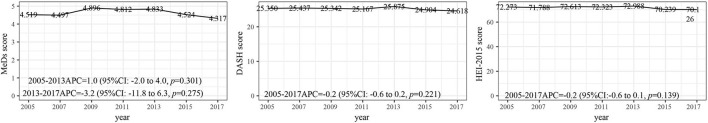
Mean dietary scores between 2005 and 2017. MEDS, Mediterranean; DASH, Dietary Approaches to Stop Hypertension; HEI-2015, Healthy Eating Index 2015; APC, annual percentage changes; CI, confidence interval.

Figure 1 describes the study design, sampling, and exclusion; our final sample included 3,190 NHANES participants, with a mean age of 62.69 (0.35) years. There were 300 individuals in the depression group and 2,868 individuals without depression. Depression is more common in women (217; 68.45%) than men. Age, sex, race, marital status, BMI, PIR, education level, sleep disorder, smoking status, pain relief prescription use, cervix cancer, ovary cancer, uterus cancer, the number of tumors, and dietary scores were found to correlate with depression (Table 1). Figures 2, 3 respectively present the depression prevalence trend and mean dietary scores in our sample. From 2005 to 2017, there was no statistical difference in depression prevalence (APC: −1.8, 95% CI: −7.6 to 4.5, *p* = 0.490).

**Table 1:** Characteristics of included participants.

**Variables**	**Total (*n =* 3,190)**	**Patients without depression (*n =* 2,868)**	**Patients with depression (*n =* 322)**	**Statistics**	***P***
**Age, mean (S.E), years**	62.69 (0.35)	63.24 (0.38)	56.71 (1.14)	T = 5.22	<0.001
**Sex**, ***n*** **(%)**				X^2^ = 8.919	0.003
Male	1,511 (43.06)	1,406 (44.11)	105 (31.55)		
Female	1,679 (56.94)	1,462 (55.89)	217 (68.45)		
**Race**, ***n*** **(%)**				X^2^ = 18.143	0.001
Mexican American	205 (2.41)	166 (2.22)	39 (4.47)		
Other Hispanic	189 (2.35)	168 (2.33)	21 (2.55)		
Non-Hispanic white	2,177 (86.51)	1,984 (87.12)	193 (79.82)		
Non-Hispanic black	459 (5.15)	412 (4.96)	47 (7.27)		
Other race-including multi-racial	160 (3.58)	138 (3.37)	22 (5.89)		
**Marital status**, ***n*** **(%)**				X^2^ = 39.868	<0.001
Married	1,831 (62.94)	1,701 (64.34)	130 (47.69)		
Widowed	529 (13.27)	472 (13.16)	57 (14.52)		
Divorced / separated	527 (14.72)	434 (13.55)	93 (27.50)		
Spinsterhood	303 (9.06)	261 (8.95)	42 (10.30)		
**BMI**, ***n*** **(%), kg/m**^**2**^				X^2^ = 13.874	<0.001
Underweight and normal	852 (28.27)	778 (28.81)	74 (22.37)		
Overweight	1,136 (34.06)	1,047 (34.67)	89 (27.37)		
Obesity	1,202 (37.67)	1,043 (36.52)	159 (50.27)		
**PIR**, ***n*** **(%)**				X^2^ = 87.376	<0.001
≤ 130%	777 (15.13)	609 (12.87)	168 (39.94)		
130%-350%	1,284 (35.55)	1,180 (35.50)	104 (36.16)		
> 350%	1,129 (49.32)	1,079 (51.64)	50 (23.91)		
**Education**, ***n*** **(%)**				X^2^ = 28.554	<0.001
≤ 11th grade (includes 12th grade with no diploma)	646 (12.08)	541 (11.12)	105 (22.59)		
High school graduate / GED equivalent	718 (21.20)	635 (20.64)	83 (27.33)		
Some college or AA degree and above	1,826 (66.72)	1,692 (68.24)	134 (50.08)		
**Sleep disorder**, ***n*** **(%)**				X^2^ = 99.113	<0.001
Yes	1,143 (38.12)	925 (34.97)	218 (72.63)		
No	2,047 (61.88)	1,943 (65.03)	104 (27.37)		
**Hypertension**, ***n*** **(%)**				X^2^ = 2.196	0.138
Yes	1,838 (51.81)	1,638 (51.38)	200 (56.54)		
No	1,352 (48.19)	1,230 (48.62)	122 (43.46)		
**Diabetes**, ***n*** **(%)**				X^2^ = 1.913	0.167
Yes	663 (16.67)	581 (16.35)	82 (20.14)		
No	2,527 (83.33)	2,287 (83.65)	240 (79.86)		
**Smoking status**, ***n*** **(%)**				X^2^ = 38.240	<0.001
Yes	1,750 (53.58)	1,534 (52.03)	216 (70.51)		
No	1,440 (46.42)	1,334 (47.97)	106 (29.49)		
**Pain relief prescription use**				X^2^ = 27.860	<0.001
No	2,321 (73.75)	2,142 (75.11)	179 (58.81)		
Yes	869 (26.25)	726 (24.89)	143 (41.19)		
**Cancer types**					
Breast cancer, *n* (%)				X^2^ = 0.739	0.390
No/unknown	2,680 (84.01)	2,409 (83.85)	271 (85.80)		
Yes	510 (15.99)	459 (16.15)	51 (14.20)		
Cervix, n (%)				X^2^ = 16.275	<0.001
No/unknown	2,966 (91.98)	2,689 (92.86)	277 (82.41)		
Yes	224 (8.02)	179 (7.14)	45 (17.59)		
Bladder, *n* (%)				X^2^ = 0.659	0.417
No/unknown	3,100 (97.88)	2,787 (97.83)	313 (98.50)		
Yes	90 (2.12)	81 (2.17)	9 (1.50)		
Colon, *n* (%)				X^2^ = 3.115	0.078
No/unknown	2,964 (94.76)	2,673 (94.96)	291 (92.51)		
Yes	226 (5.24)	195 (5.04)	31 (7.49)		
Kidney, *n* (%)				X^2^ = 0.255	0.613
No/unknown	3,127 (98.49)	2,813 (98.52)	314 (98.15)		
Yes	63 (1.51)	55 (1.48)	8 (1.85)		
Melanoma, *n* (%)				X^2^ = 0.440	0.507
No/unknown	2,971 (91.38)	2,664 (91.24)	307 (92.94)		
Yes	219 (8.62)	204 (8.76)	15 (7.06)		
Ovary, *n* (%)				X^2^ = 10.051	0.002
No/unknown	3,101 (97.41)	2,802 (97.68)	299 (94.40)		
Yes	89 (2.59)	66 (2.32)	23 (5.60)		
Prostate, *n* (%)				X^2^ = 2.696	0.101
No/unknown	2,669 (89.70)	2,379 (89.40)	290 (92.92)		
Yes	521 (10.30)	489 (10.60)	32 (7.08)		
Skin, *n* (%)				X^2^ = 1.869	0.172
No/unknown	2,387 (67.60)	2,126 (67.10)	261 (73.02)		
Yes	803 (32.40)	742 (32.90)	61 (26.98)		
Uterus, n (%)				X^2^ = 7.310	0.007
No/unknown	3,048 (95.95)	2,756 (96.30)	292 (92.13)		
Yes	142 (4.05)	112 (3.70)	30 (7.87)		
The number of tumors, *n* (%)				X^2^ = 5.018	0.025
One	2,863 (89.55)	2,587 (89.94)	276 (85.29)		
Two or more	327 (10.45)	281 (10.06)	46 (14.71)		
**Dietary scores**					
MEDS, mean (S.E)	4.63 (0.06)	4.71 (0.06)	3.72 (0.19)	T = 5.15	<0.001
DASH, mean (S.E)	25.22 (0.12)	25.31 (0.12)	24.23 (0.29)	T = 3.63	<0.001
HEI 2015, mean (S.E)	71.68 (0.27)	71.93 (0.27)	68.97 (0.74)	T = 3.91	<0.001

PIR, property income ratio; GED, general equivalency diploma; MEDS, Mediterranean; DASH, Dietary Approaches to Stop Hypertension; HEI, Healthy Eating Index.

### Correlation between MEDS, DASH, HEI 2015, and depression

From models 1 and 2, we could notice that MEDS, DASH, and HEI were all correlated with depression in patients with cancer. After adjusting for sex, age, race, smoking history, sleep disorders, education level, family poverty ratio, marital status, BMI, cervix cancer, ovary cancer, uterus cancer, pain medication use, and the number of different types of cancer, only MEDS showed a link to a decreased risk of depression in patients with cancer [odds ratio: (OR): 0.90, 95% confidence interval (CI): 0.82–0.97, *p* = 0.010]. The significant correlations between diet scores and depression are shown in Table 2.

**Table 2:** Association analysis of MEDS, DASH, and HEI 2015 with depression.

**Diet scores**	**Model 1**		**Model 2**		**Model 3**	
	**OR (95%CI)**	***P***	**OR (95%CI)**	***P***	**OR (95%CI)**	***P***
MEDS	0.81 (0.74–0.88)	<0.001	0.83 (0.77–0.90)	<0.001	0.90 (0.82–0.97)	0.010
DASH	0.94 (0.91–0.98)	<0.001	0.95 (0.92–0.98)	<0.001	0.99 (0.95–1.03)	0.562
HEI	0.97 (0.96–0.99)	<0.001	0.98 (0.96–0.99)	0.001	0.99 (0.98–1.01)	0.557

MEDS, Mediterranean; DASH, Dietary Approaches to Stop Hypertension; HEI, Healthy Eating Index; OR, odds ratio; CI, confidence interval; model 1 was an unadjusted model; model 2 was adjusted for sex, age, and race; and model 3 was adjusted for sex, age, ethnicity, smoking history, sleep disorders, education level, family poverty ratio, marital status, BMI, cervix cancer, ovary cancer, uterus cancer, pain relief prescription, and number of different types of cancer.

### Correlation between MEDs, DASH, HEI 2015, and depression among different population

The results of the subgroup analysis of sleep disorders suggested that MEDS scores were correlated with depression in cancer patients with sleep disorders (OR: 0.84, 95% CI: 0.76–0.93, *p* = 0.001; Table 3).

**Table 3:** Association analysis of dietary scores MEDS, DASH, HEI2015, and depression in sleep disorder subgroup.

**Population**	**Dietary scores**	**Model 1**		**Model 2**		**Model 3**	
		**OR (95%CI)**	***P***	**OR (95%CI)**	***P***	**OR (95%CI)**	***P***
Cancer survivor with sleep disorder							
	MEDS	0.79 (0.71–0.87)	<0.001	0.81 (0.74–0.90)	<0.001	0.84 (0.76–0.93)	0.001
	DASH	0.94 (0.90–0.98)	0.002	0.95 (0.91–0.99)	0.019	0.98 (0.93–1.03)	0.353
	HEI	0.97 (0.95–0.99)	0.003	0.98 (0.95–0.99)	0.015	0.99 (0.97–1.01)	0.355
Cancer survivor without sleep disorder							
	MEDS	0.91 (0.80–1.03)	0.134	0.93 (0.82–1.05)	0.249	1.00 (0.88–1.14)	0.987
	DASH	0.97 (0.91–1.02)	0.205	0.96 (0.91–1.02)	0.221	1.01 (0.95–1.07)	0.714
	HEI	0.99 (0.96–1.00)	0.236	0.99 (0.97–1.01)	0.322	1.01 (0.99–1.03)	0.530

OR, odds ratio; CI, confidence interval; MEDS, Mediterranean; DASH, Dietary Approaches to Stop Hypertension; HEI, Healthy Eating Index; model 1 was an unadjusted model; model 2 was adjusted for sex, age, and race; and model 3 was adjusted for sex, age, ethnicity, smoking history, sleep disorders, education level, family poverty ratio, marital status, BMI, cervix cancer, ovary cancer, uterus cancer, pain relief prescription, and number of different types of cancer.

Based on sex subgroup analysis, we found that the MEDS (OR: 0.83, 95% CI: 0.74–0.92, *p* < 0.001) and the DASH (OR: 0.95, 95% CI: 0.91–0.99, *p* = 0.018) were correlated with a decreased risk of depression in female patients with cancer. Moreover, the MEDS pattern may lower the risk of depression in women than the DASH pattern with a standardized beta being −19.16 (Table 4).

**Table 4:** Association analysis of dietary scores and depression in sex subgroups.

**Sex**	**Dietary scores**	**Model 1**		**Model 2**		**Model 3**		
		**OR (95%CI)**	***P***	**Or (95%CI)**	***P***	**OR (95%CI)**	***P***	**Standardized β**
Male								
	MEDS	0.92 (0.81–1.04)	0.171	0.93 (0.82–1.05)	0.217	1.01 (0.90–1.14)	0.838	
	DASH	1.02 (0.95–1.09)	0.597	1.03 (0.96–1.10)	0.420	1.06 (0.99–1.14)	0.095	
	HEI	0.99 (0.97–1.02)	0.539	0.99 (0.97–1.02)	0.788	1.01 (0.98–1.03)	0.621	
Female								
	MEDS	0.78 (0.71–0.86)	<0.001	0.78 (0.71–0.87)	<0.001	0.83 (0.74–0.92)	<0.001	−19.16
	DASH	0.89 (0.86–0.93)	<0.001	0.91 (0.87–0.94)	<0.001	0.95 (0.91–0.99)	0.018	−9.91
	HEI	0.96 (0.94–0.98)	<0.001	0.97 (0.95–0.98)	<0.001	0.99 (0.97–1.01)	0.303	

OR, odds ratio; CI, confidence interval; MEDS, Mediterranean; DASH, Dietary Approaches to Stop Hypertension; HEI, Healthy Eating Index; model 1 was an unadjusted model; model 2 was adjusted for age, and race; and model was 3 adjusted for age, ethnicity, smoking history, sleep disorders, education level, family poverty ratio, marital status, BMI, pain relief prescription, and number of different types of cancer.

According to the female reproductive system cancer subgroup analysis, MEDS diet (OR: 0.69, 95% CI: 0.57–0.82, *p* < 0.001), DASH (OR: 0.90, 95% CI: 0.84–0.96, *p* = 0.002), and HEI score (OR: 0.96, 95% CI: 0.93–0.99, *p* = 0.005) were all related to a lower risk of depression in female reproductive system patients with cancer. However, concerning standardized beta values, the MEDS diet was correlated with a lower risk of depression in women with female reproductive cancer (Table 5).

**Table 5:** Association analysis of dietary scores and depression in female genital cancer subgroup.

**Population**	**Dietary scores**	**Model 1**		**Model 2**		**Model 3**		
		**OR (95%CI)**	***P***	**OR (95%CI)**	***P***	**OR (95%CI)**	***P***	**Standardized β**
Women with female genital cancer								
	MEDS	0.69 (0.58–0.82)	<0.001	0.68 (0.57–0.81)	<0.001	0.69 (0.57–0.82)	<0.001	−36.32
	DASH	0.88 (0.83–0.93)	<0.001	0.91 (0.85–0.97)	0.003	0.90 (0.84–0.96)	0.002	−19.10
	HEI	0.94 (0.92–0.97)	<0.001	0.95 (0.92–0.98)	<0.001	0.96 (0.93–0.99)	0.005	−20.22
Women without female genital cancer								
	MEDS	0.85 (0.76–0.95)	0.003	0.85 (0.76–0.96)	0.006	0.90 (0.79–1.03)	0.128	
	DASH	0.91 (0.86–0.96)	<0.001	0.92 (0.87–0.96)	<0.001	0.97 (0.92–1.02)	0.257	
HEI	HEI	0.97 (0.95–0.99)	0.013	0.98 (0.95–1.00)	0.053	1.01 (0.98–1.03)	0.509	

OR, odds ratio; CI, confidence interval; MEDS, Mediterranean; DASH, Dietary Approaches to Stop Hypertension; HEI, Healthy Eating Index; model 1 was an unadjusted model; model 2 adjusted for age, and race; and model 3 adjusted for age, ethnicity, smoking history, sleep disorders, education level, family poverty ratio, marital status, BMI, pain relief prescription, and number of different types of cancer.

Regarding the number of tumors subgroup analysis, the MEDS score still was correlated with a decreased risk of depression in patients with only 1 cancer (OR: 0.90, 95% CI: 0.82–0.98, *p* = 0.019; Table 6).

**Table 6:** Association analysis of dietary scores and depression in the number of tumors subgroup.

**Numbers of tumors**	**Dietary scores**	**Model 1**		**Model 2**		**Model 3**	
		**OR (95%CI)**	***P***	**OR (95%CI)**	***P***	**OR (95%CI)**	***P***
One							
	MEDS	0.81 (0.74–0.88)	<0.001	0.83 (0.76–0.90)	<0.001	0.90 (0.82–0.98)	0.019
	DASH	0.94 (0.91–0.98)	0.002	0.95 (0.91–0.98)	0.006	1.00 (0.96–1.05)	0.890
	HEI	0.97 (0.96–0.99)	<0.001	0.98 (0.96–0.99)	<0.001	1.00 (0.98–1.02)	0.992
Two or more							
	MEDS	0.83 (0.69–0.99)	0.041	0.88 (0.71–1.08)	0.202	0.91 (0.75–1.09)	0.299
	DASH	0.94 (0.88–0.99)	0.032	0.97 (0.91–1.03)	0.337	0.95 (0.88–1.01)	0.102
	HEI	0.97 (0.93–1.01)	0.089	0.98 (0.94–1.03)	0.462	0.99 (0.95–1.03)	0.495

OR, odds ratio; CI, confidence interval; MEDS, Mediterranean; DASH, Dietary Approaches to Stop Hypertension; HEI, Healthy Eating Index; model 1 was an unadjusted model; model 2 was adjusted for sex, age, and race; and model 3 was adjusted for sex, age, ethnicity, smoking history, sleep disorders, education level, family poverty ratio, marital status, BMI, cervix cancer, ovary cancer, uterus cancer, and pain relief prescription.

Concerning whether or not to use pain relief prescription subgroup analysis and pain relief prescription use, MEDS score (OR: 0.86, 95% CI: 0.86–0.98, *p* = 0.024) and DASH (OR: 0.91, 95% CI: 0.84–0.98, *p* = 0.015) score decreased the risk of depression in patients with cancer using pain relief prescription (Table 7).

**Table 7:** Association analysis of dietary scores and depression in whether use pain relief prescription subgroup.

**Treatment**	**Dietary scores**	**Model 1**	**Model 2**	**Model 3**				
		**OR (95%CI)**	***P***	**OR (95%CI)**	***P***	**OR (95%CI)**	***P***	**Standardized** β
No pain relief prescription								
	MEDS	0.85 (0.76–0.94)	0.003	0.86 (0.77–0.95)	0.005	0.91 (0.81–1.01)	0.074	
	DASH	0.97 (0.94–1.01)	0.186	0.98 (0.94–1.02)	0.374	1.03 (0.98–1.08)	0.252	
	HEI	0.98 (0.96–0.99)	0.022	0.98 (0.97–1.00)	0.082	1.00 (0.98–1.02)	0.793	
Pain relief prescription								
	MEDS	0.78 (0.69–0.88)	<0.001	0.82 (0.73–0.94)	0.003	0.86 (0.86–0.98)	0.024	−14.30
	DASH	0.90 (0.85–0.96)	0.001	0.91 (0.85–0.98)	0.010	0.91 (0.84–0.98)	0.015	−16.98
	HEI	0.96 (0.94–0.99)	0.002	0.97 (0.95–0.99)	0.018	0.98 (0.96–1.01)	0.204	

OR, odds ratio; CI, confidence interval; MEDS, Mediterranean; DASH, Dietary Approaches to Stop Hypertension; HEI, Healthy Eating Index; model 1 was an unadjusted model; model 2 was adjusted for sex, age, and race; and model 3 was adjusted for sex, age, ethnicity, smoking history, sleep disorders, education level, family poverty ratio, marital status, BMI, cervix cancer, ovary cancer, uterus cancer, and number of the cancer.

## Discussion

Based on the NHANES database, several studies have assessed the impact of lifestyle on an individual's health (Del Giudice et al., 2021; Glover et al., 2022a,b). A growing body of research suggests a protective role of a good diet style against depression (Winpenny et al., 2018; Gibson-Smith et al., 2020; Oliván-Blázquez et al., 2021); nevertheless, evidence to evaluate this correlation among patients with cancer is scarce. In this study, we assessed the correlation between diet scores and depression among patients with cancer. From our findings, the MEDS score showed a link to a decreased risk of depression in patients with cancer. Moreover, MEDS score was correlated with depression in cancer patients with sleep disorders, in female patients with cancer, and particularly in female cancer reproductive system patients. MEDS score and DASH score also showed a decreased risk of depression in patients using pain relief prescriptions.

In this study, the MEDS score showed a link to a decreased risk of depression in patients with cancer. Increasing evidence implicates certain dietary patterns, such as higher intake of fruit, vegetables, and fish, as being beneficial to brain health (Hossain et al., 2020). The MEDs diet is receiving significant attention as regards its role in preserving cognitive health and protecting against depression (Román et al., 2019). This diet is typically characterized by higher intakes of fruit, vegetables, whole grains, fish, unsaturated fatty acids, and regular but moderate consumption of alcohol (Moore et al., 2018). Better post-diagnosis diet quality has been found to be associated with less cancer-related fatigue in breast cancer survivors (George et al., 2014). A study demonstrated that post-diagnosis diet quality was directly associated with subsequent mental and physical functioning in breast cancer survivors (Wayne et al., 2006). Diet quality may be an important factor influencing the manifestation of depressive symptoms in patients with cancer or conversely, poorer diet quality may be an outcome of depression. Thus, for patients with cancer, a healthy diet, such as the MEDS diet, may be beneficial to improve mental health, reduce the risk of depression, and improve life wellbeing.

Based on our subgroup analysis, MEDS scores were correlated with depression in female patients with cancer, particularly in female cancer reproductive system patients. Yin et al. (2021) found that higher adherence to a MEDS diet in middle age was associated with a lower risk of depression later in life among Swedish women. Wu et al. (2020) demonstrated that acid-producing diets were associated with depression; the correlations were stronger in women who had breast cancer diagnosed before age 55. Women with cancer of the reproductive system should pay more attention to a high-quality diet and eat more vegetables, fruits, whole grains, fish, and unsaturated fatty acids to reduce the risk of depression. In this study, we did not find a correlation between diet scores and depression risk in male patients with cancer. Differences in hormones between men and women may be a possible reason for this difference. A systematic review has found a correlation between male infertility and overall male health (Del Giudice et al., 2020).

In our findings, MEDS score and DASH score were associated with a decreased risk of depression in patients with cancer using pain relief prescriptions. A previous study showed a strong correlation between depression and cancer pain where the odds of having a major depression are more than 4 times higher in those with pain complaints (Alemayehu et al., 2018). Cancer pain is caused either directly by the tumor (primary tumor invasion or metastases) or indirectly by cancer treatments (surgery, chemotherapy, and radiotherapy) (Caraceni and Shkodra, 2019). A previous study (Maggi et al., 2019) assessing the psychological impact of different primary treatments for prostate cancer reported a mutual influence between functional and psychological modifications induced by treatments. Vartolomei et al. (2022) found that patients with small renal masses that underwent active surveillance seem to have a lower quality of life with respect to physical activity than their counterparts that underwent active treatment with some additional degree of psychological distress. The association between diet scores and depression in cancer patients with different treatments that include pain relief prescriptions needs further investigation.

There are several hypotheses regarding the possible etiological basis for this positive effect of the MEDs score on the risk of depression. Firstly, the high levels of antioxidants (fiber, β-carotenes, and vitamins A, C, D, and E) in fruits and vegetables have beneficial effects on depression (Sánchez-Villegas et al., 2015; Liu et al., 2016). Second, vitamin B6, vitamin B12, and folate are involved in neurochemical pathways, and the synthesis of monoamines, such as serotonin and noradrenaline, is related to the risk of depression (Sánchez-Villegas et al., 2009). Third, the gastrointestinal microbiome plays an important role in mental health. It is involved in two-way communication between the brain and the gastrointestinal tract *via* the gut-brain axis, which may be the fundamental link between the microbiome and depression (Taylor and Holscher, 2020). Dietary fiber deficiency leads to a significant loss of gut microbiome diversity and affects the function of gut bacteria (Cheung et al., 2019). It was subsequently reported that dietary fiber intake from fruits and vegetables was negatively associated with depressive symptoms (Cheung et al., 2019).

Some of the limitations of this article cannot be ignored. First, a correlation derived from this cross-sectional study does not necessarily indicate causality regarding the relationship between dietary patterns and depression in patients with cancer. Some mental illnesses might influence appetite and alter an individual's food choices; therefore, poor diet quality may be the result of mental health symptoms, rather than a causative factor. Second, we assessed depression with a self-report rating scale, rather than definite cases of depression based on a clinician-administered structured diagnostic interview. In addition, due to the limitations of the database, the time of diagnosis of the tumor and the severity of the tumor was not available. Prospective studies would be highly informative to further assess the association between diet scores and the risk of depression in patients with cancer.

## Conclusions

Mediterranean score was correlated with a reduced risk of depression in patients with cancer. However, additional studies are required before the results can be generalized and clinical recommendations can be given.

## Data availability statement

Publicly available datasets were analyzed in this study. This data can be found here: NHANES database, Available online at: https://wwwn.cdc.gov/nchs/nhanes/.

## Ethics statement

Ethical approval was not provided for this study on human participants because the data was accessed from NHANES (a publicly available database). The patients/participants provided their written informed consent to participate in this study.

## Author contributions

NX and QA designed the study and collected, analyzed, and interpreted the data. NX wrote the manuscript. QA critically reviewed, edited, and approved the manuscript. All authors read and approved the final manuscript.

## Funding

This study was supported by Jiangsu Cancer Hospital 2021 Science and Technology Development Fund project truth-seeking clinical research special project (ZL202111).

## Conflict of interest

The authors declare that the research was conducted in the absence of any commercial or financial relationships that could be construed as a potential conflict of interest.

## Publisher's note

All claims expressed in this article are solely those of the authors and do not necessarily represent those of their affiliated organizations, or those of the publisher, the editors and the reviewers. Any product that may be evaluated in this article, or claim that may be made by its manufacturer, is not guaranteed or endorsed by the publisher.

## References

[B1] Akimana B. Abbo C. Balagadde-Kambugu J. Nakimuli-Mpungu E. (2019). Prevalence and factors associated with major depressive disorder in children and adolescents at the Uganda Cancer Institute. BMC Cancer 19, 466. 10.1186/s12885-019-5635-z 31101016 PMC6525350

[B2] Alemayehu M. Deyessa N. Medihin G. Fekadu A. (2018). A descriptive analysis of depression and pain complaints among patients with cancer in a low income country. PLoS ONE 13, e0193713. 10.1371/journal.pone.0193713 29513716 PMC5841758

[B3] American Diabetes Association. (2019). Standards of medical care in diabetes-2019 abridged for primary care providers. Clin. Diab. 37, 11–34. 10.2337/cd18-0105 30705493 PMC6336119

[B4] Belladelli F. Del Giudice F. Kasman A. Salonia A. Eisenberg M. L. (2021). The association between testosterone, estradiol and their ratio and mortality among US men. Andrologia 53, e13993. 10.1111/and.13993 33666951

[B5] Caraceni A. Shkodra M. (2019). Cancer pain assessment and classification. Cancers 11, 510. 10.3390/cancers11040510 30974857 PMC6521068

[B6] Cheung S. G. Goldenthal A. R. Uhlemann A. C. Mann J. J. Miller J. M. Sublette M. E. (2019). Systematic review of gut microbiota and major depression. Front. Psychiatry 10, 34. 10.3389/fpsyt.2019.00034 30804820 PMC6378305

[B7] De La Cruz N. Shabaneh O. Appiah D. (2021). The Association of Ideal Cardiovascular Health and Ocular Diseases Among US Adults. Am. J. Med. 134, 252–9.e251. 10.1016/j.amjmed.2020.06.004 32828726

[B8] Del Giudice F. Glover F. Belladelli F. De Berardinis E. Sciarra A. Salciccia S.. (2021). Association of daily step count and serum testosterone among men in the United States. Endocrine 72, 874–881. 10.1007/s12020-021-02631-2 33580402 PMC8159788

[B9] Del Giudice F. Kasman A. M. Ferro M. Sciarra A. De Berardinis E. Belladelli F.. (2020). Clinical correlation among male infertility and overall male health: A systematic review of the literature. Investig. Clin. Urol. 61, 355–371. 10.4111/icu.2020.61.4.355 32665992 PMC7329649

[B10] Erim D. O. Bensen J. T. Mohler J. L. Fontham E. T. H. Song L. Farnan L.. (2019). Prevalence and predictors of probable depression in prostate cancer survivors. Cancer 125, 3418–3427. 10.1002/cncr.32338 31246284 PMC7465428

[B11] George S. M. Alfano C. M. Neuhouser M. L. Smith A. W. Baumgartner R. N. Baumgartner K. B.. (2014). Better postdiagnosis diet quality is associated with less cancer-related fatigue in breast cancer survivors. J. Cancer Surviv. 8, 680–687. 10.1007/s11764-014-0381-3 25001403 PMC6993811

[B12] Gibson-Smith D. Bot M. Brouwer I. A. Visser M. Giltay E. J. Penninx B. (2020). Association of food groups with depression and anxiety disorders. Eur. J. Nutr. 59, 767–778. 10.1007/s00394-019-01943-4 30945032 PMC7058560

[B13] Glover F. E. Caudle W. M. Del Giudice F. Belladelli F. Mulloy E. Lawal E.. (2022a). The association between caffeine intake and testosterone: NHANES 2013-2014. Nutr. J. 21, 33. 10.1186/s12937-022-00783-z 35578259 PMC9112543

[B14] Glover F. E. Del Giudice F. Belladelli F. Ryan P. B. Chen T. Eisenberg M. L.. (2022b). The association between 2,4-D and serum testosterone levels: NHANES 2013-2014. J. Endocrinol. Invest. 45, 787–796. 10.1007/s40618-021-01709-y 34837643

[B15] Hossain S. Beydoun M. A. Weiss J. Kuczmarski M. F. Evans M. K. Zonderman A. B. (2020). Longitudinal associations between dietary quality and Alzheimer's disease genetic risk on cognitive performance among African American adults. Br. J. Nutr. 124, 1264–1276. 10.1017/S0007114520001269 32248879 PMC7541564

[B16] Krebs-Smith S. M. Pannucci T. E. Subar A. F. Kirkpatrick S. I. Lerman J. L. Tooze J. A.. (2018). Update of the Healthy Eating Index: HEI-2015. J. Acad. Nutr. Diet. 118, 1591–1602. 10.1016/j.jand.2018.05.021 30146071 PMC6719291

[B17] Kroenke K. Spitzer R. L. Williams J. B. (2001). The PHQ-9: validity of a brief depression severity measure. J. Gen. Intern. Med. 16, 606–613. 10.1046/j.1525-1497.2001.016009606.x 11556941 PMC1495268

[B18] Le L. Yu L. Guan C. Zhang X. (2018). Epidemiology, etiology, screening, psychotherapy of malignant tumor patients with secondary depressive disorder. Curr. Pharm. Des. 24, 2591–2596. 10.2174/1381612824666180727125448 30051784

[B19] Lee S. J. Lee K. W. Cho M. S. (2021). Association of food insecurity with nutrient intake and depression among korean and US adults: data from the 2014 Korea and the 2013-2014 US national health and nutrition examination surveys. Int. J. Environ. Res. Public Health 18, 506. 10.3390/ijerph18020506 33435492 PMC7827165

[B20] Li Z. Wang W. Xin X. Song X. Zhang D. (2018). Association of total zinc, iron, copper and selenium intakes with depression in the US adults. J. Affect. Disord. 228, 68–74. 10.1016/j.jad.2017.12.004 29232566

[B21] Liu X. Yan Y. Li F. Zhang D. (2016). Fruit and vegetable consumption and the risk of depression: A meta-analysis. Nutrition 32, 296–302. 10.1016/j.nut.2015.09.009 26691768

[B22] Maggi M. Gentilucci A. Salciccia S. Gatto A. Gentile V. Colarieti A.. (2019). Psychological impact of different primary treatments for prostate cancer: A critical analysis. Andrologia 51, e13157. 10.1111/and.13157 30281167

[B23] Miller K. D. Nogueira L. Mariotto A. B. Rowland J. H. Yabroff K. R. Alfano C. M.. (2019). Cancer treatment and survivorship statistics, 2019. CA Cancer J. Clin. 69, 363–385. 10.3322/caac.21565 31184787

[B24] Moore K. Hughes C. F. Ward M. Hoey L. Mcnulty H. (2018). Diet, nutrition and the ageing brain: current evidence and new directions. Proc. Nutr. Soc. 77, 152–163. 10.1017/S0029665117004177 29316987

[B25] National Health Nutrition Examination Survey. (2020). Available online at: https://www.cdc.gov/nchs/nhanes/about_nhanes.htm (accessed June 15, 2020).

[B26] Oliván-Blázquez B. Aguilar-Latorre A. Motrico E. Gómez-Gómez I. Zabaleta-Del-Olmo E. Couso-Viana S.. (2021). The relationship between adherence to the mediterranean diet, intake of specific foods and depression in an adult population (45-75 Years) in primary health care. a cross-sectional descriptive study. Nutrients 13, 2724. 10.3390/nu13082724 34444884 PMC8399773

[B27] Perez-Cornago A. Sanchez-Villegas A. Bes-Rastrollo M. Gea A. Molero P. Lahortiga-Ramos F.. (2017). Relationship between adherence to Dietary Approaches to Stop Hypertension (DASH) diet indices and incidence of depression during up to 8 years of follow-up. Public Health Nutr. 20, 2383–2392. 10.1017/S1368980016001531 27335121 PMC10261567

[B28] Rahimlou M. Morshedzadeh N. Karimi S. Jafarirad S. (2018). Association between dietary glycemic index and glycemic load with depression: a systematic review. Eur. J. Nutr. 57, 2333–2340. 10.1007/s00394-018-1710-5 29744611

[B29] Robson M. J. Quinlan M. A. Blakely R. D. (2017). Immune system activation and depression: roles of serotonin in the central nervous system and periphery. ACS Chem. Neurosci. 8, 932–942. 10.1021/acschemneuro.6b00412 28345868

[B30] Román G. C. Jackson R. E. Gadhia R. Román A. N. Reis J. (2019). Mediterranean diet: The role of long-chain ω-3 fatty acids in fish; polyphenols in fruits, vegetables, cereals, coffee, tea, cacao and wine; probiotics and vitamins in prevention of stroke, age-related cognitive decline, and Alzheimer disease. Rev. Neurol. 175, 724–741. 10.1016/j.neurol.2019.08.005 31521398

[B31] Sakai H. Murakami K. Kobayashi S. Suga H. Sasaki S. (2017). Food-based diet quality score in relation to depressive symptoms in young and middle-aged Japanese women. Br. J. Nutr. 117, 1674–1681. 10.1017/S0007114517001581 28789727

[B32] Sánchez-Villegas A. Doreste J. Schlatter J. Pla J. Bes-Rastrollo M. Martínez-González M. A. (2009). Association between folate, vitamin B(6) and vitamin B(12) intake and depression in the SUN cohort study. J. Hum. Nutr. Diet 22, 122–133. 10.1111/j.1365-277X.2008.00931.x 19175490

[B33] Sánchez-Villegas A. Martínez-González M. A. Estruch R. Salas-Salvad,ó J. Corella D. Covas M. I.. (2013). Mediterranean dietary pattern and depression: the PREDIMED randomized trial. BMC Med. 11, 208. 10.1186/1741-7015-11-208 24229349 PMC3848350

[B34] Sánchez-Villegas A. Ruíz-Canela M. De La Fuente-Arrillaga C. Gea A. Shivappa N. Hébert J. R.. (2015). Dietary inflammatory index, cardiometabolic conditions and depression in the Seguimiento Universidad de Navarra cohort study. Br. J. Nutr. 114, 1471–1479. 10.1017/S0007114515003074 26344165

[B35] Siegel R. L. Miller K. D. Fuchs H. E. Jemal A. (2021). Cancer Statistics, 2021. CA Cancer J. Clin. 71, 7–33. 10.3322/caac.21654 33433946

[B36] Steele C. C. Steele T. J. Rosenkranz S. K. Lee J. Ade C. J. (2021). Health behaviors and patient-practitioner communication in cancer patients and the general population: an analysis of the National Health and Nutrition Examination Survey (NHANES) 2005-2014. Supp. Care Cancer 29, 3877–3884. 10.1007/s00520-020-05940-w 33389166

[B37] Taylor A. M. Holscher H. D. (2020). A review of dietary and microbial connections to depression, anxiety, and stress. Nutr. Neurosci. 23, 237–250. 10.1080/1028415X.2018.1493808 29985786

[B38] Vartolomei L. Cotru,? A. Stanciu C. Delcea C. Tozzi M. Lievore E.. (2022). Quality of life and psychological distress among patients with small renal masses. J. Clin. Med. 11, 3944. 10.3390/jcm11143944 35887708 PMC9324284

[B39] Viale P. H. (2020). The American cancer society's facts and figures: 2020 edition. J. Adv. Pract. Oncol. 11, 135–136. 10.6004/jadpro.2020.11.2.1 33532112 PMC7848816

[B40] Wang K. Zhao Y. Nie J. Xu H. Yu C. Wang S. (2021). Higher HEI-2015 score is associated with reduced risk of depression: result from NHANES 2005-2016. Nutrients 13, 348. 10.3390/nu13020348 33503826 PMC7911826

[B41] Wayne S. J. Baumgartner K. Baumgartner R. N. Bernstein L. Bowen D. J. Ballard-Barbash R. (2006). Diet quality is directly associated with quality of life in breast cancer survivors. Breast Cancer Res. Treat. 96, 227–232. 10.1007/s10549-005-9018-6 16538543

[B42] Winpenny E. M. Van Harmelen A. L. White M. Van Sluijs E. M. Goodyer I. M. (2018). Diet quality and depressive symptoms in adolescence: no cross-sectional or prospective associations following adjustment for covariates. Public Health Nutr. 21, 2376–2384. 10.1017/S1368980018001179 29766837 PMC6137369

[B43] Wu T. Hsu F. C. Pierce J. P. (2020). Acid-producing diet and depressive symptoms among breast cancer survivors: a longitudinal study. Cancers 12, 3183. 10.3390/cancers12113183 33138152 PMC7692146

[B44] Yin W. Löf M. Chen R. Hultman C. M. Fang F. Sandin S. (2021). Mediterranean diet and depression: a population-based cohort study. Int. J. Behav. Nutr. Phys. Act. 18, 153. 10.1186/s12966-021-01227-3 34838037 PMC8627099

